# Implant-abutment screw removal torque values between customized titanium abutment, straight titanium abutment, and hybrid zirconia abutment after a million cyclic loading: an in vitro comparative study

**DOI:** 10.1186/s40729-021-00378-z

**Published:** 2021-10-04

**Authors:** Disayut Klongbunjit, Weerapan Aunmeungtong, Pathawee Khongkhunthian

**Affiliations:** Center of Excellence for Dental Implantology, Faculty of Dentistry, Chiang Mai University, Suthep, A. Muang, Chiang Mai, 50200 Thailand

**Keywords:** Bending moment, Removal torque, Cyclic loading, Abutment

## Abstract

**Purpose:**

The aim of this study was to compare removal torque values after mechanical cyclic loading and bending moment after the static compression testing of customized titanium abutment compared with prefabricated and hybrid abutments.

**Materials and methods:**

The study was developed according to ISO 14801:2016. Sixty implants were divided into three groups equally: Straight titanium abutment group, Customized titanium abutment group, and Hybrid zirconia abutment group. Abutments were fabricated with zirconia restoration. Forty five implants underwent for cyclic loading. The removal torque values were measured after a fatigue test was conducted at 0 cycles (control), 50,000 cycles and 1,000,000 cycles. In the second experiment, 15 implants were divided into the same groups. Then, bending moments were investigated.

**Results:**

The mean initial removal torque value was significantly higher than 50,000 cycles and 1,000,000 cycles (*P* < 0.001). The comparison of mean removal torque value between types of abutments was not significantly different (*P* > 0.05), and the bending moments of all abutments were not significantly different (*P* > 0.05).

**Conclusions:**

From the boundary of this in-vitro study, it could be concluded that customized titanium abutment and hybrid abutment were not significantly different in terms of removal torque values after the fatigue test. The bending moment between types of abutment were not significantly different. Thus, it could be concluded that abutment type does not significantly influence abutment stability or fracture strength.

## Background

A meta-analysis reported the survival rate of an implant-supported single crown at 5 years was 97.2% and at 10 years was 95.2% [1]. The biological and technical complications of implant-supported single crown were investigated. The most biological complication reported is peri-implant mucosal lesion [1, 2]. Mechanical complications, such as fracture of material, loss of retention, and screw loss are reported [1, 3]. The most common mechanical complication is screw loosening. The cumulative incidence rate of screw loosening over 5 years of observation was 8.8% [1]. Screw loosening refers to the unwanted rotation of an implant screw in a counterclockwise direction. This is a risk factor of screw fracture [4]. Screw loosening may result from excessive bite forces and non-functional loading [5].

The mechanism of an abutment screw to retain an abutment and fixture together depends on the mechanical properties of the screw. When a screw is tightened, it elongates and generates force, which is called “clamping force”. The force results from the elastic recovery of screw material. Tightening force is called “preload”. Decreased separating force and increased clamping force stabilize the abutment connection and prevent screw loosening [6]. The settling effect is preload loss in 2–3 min to 15 h after screw tightening without any external force intervention [7]. The settling effect could influence preload loss by 2–10% [5]. Siamos et al. recommended a protocol to prevent the settling effect, suggesting that the screw should be retightened 10 min after first-load application [8].

When the external load is beyond the preload, abutment-screw connection loses stability, affecting the vibration and micromovement of the interfaces, which causes screw loosening [5]. Huang et al. summarized the various factors that affect abutment-screw loosening in implant-retained prostheses. Abutment geometry and the manufacturing method influence the stability of the implant-screw connection [7]. El-Sheikh et al. reported that expanding the angulation and length of the collar increases the risk of screw loosening. The length of an abutment functions as a vertical cantilever, which gives bending force to the implant screw by the principle of levers [9]. Taper contact design makes friction-lock mechanism, retention results from the frictional resistance through a Morse taper design, which also stabilizes the connection [10]. Abutment fabrication influences the incidence of screw loosening. Kano et al. reported that a machined abutment has less incidence of torque loss compared with a cast abutment [11]. This screw joint stability results from the smaller gap between the abutment and fixture interface compared with a machined abutment [12]. Recently, some abutments use an anodized technique to colour abutments. However, this procedure influences abutment stability, which is significantly reduced by about 20% removal torque [13].

The implant abutment could be classified according to implant-abutment connection, abutment material, retention with prosthesis, and method of fabrication [14]. According to manufacture criteria, abutments are classified into customized abutment and prefabricated (stock) abutment [14, 15]. Most prefabricated abutments are made from titanium. Manufacturers offer them in straight and angled types. However, to achieve good emergence profile and aesthetics, dentists should place the implant in a precise position and angulation [15]. Customized abutments provide an individual emergence profile. A dentist can position the margin of a crown according to the location of the soft tissue margin [14]. Computer-aided design/computer-assisted manufacture (CAD/CAM) technology is applied for manufacturing customized abutments [16]. Customized titanium abutments with CAD/CAM technology are suggested as the standard choice due to their high clinical success rates and less corrosion than a universal castable long abutment (UCLA abutment) [17]. However, the grey colour of a titanium abutment shines through in thin soft tissue areas, and a customized titanium abutment selection is an aesthetic risk option [18]. To minimize the metal colour of titanium abutments, a zirconia framework is used due to its aesthetic qualities and biocompatibility. A zirconia framework is made up over a titanium neck. This system is called a “hybrid zirconia abutment” or “ti-base abutment” [19].

Murphy et al. reported that the teeth contact only 5.9% of entire occlusal stroke [20]. Chewing cycle simulation in a laboratory could represent clinical oral function. According to a study from Outhwaite et al., one million cycles represent a clinical chewing cycle of about 5 years [21]. Sakaguchi and colleagues reported that 1,250,000 cycles is equivalent to 5 years [22]. The results could be different, because the frequency of chewing cycles ranges from 1 to 19 Hz [23]. According to a study from Simon et al. [24], the incidence of abutment screw loosening in premolar and molar region was 7.4%. These areas carry a maximum vertical loading force of approximately 120–150 N [25]. Benjaboonyazit et al. reported that removal torque values decrease significantly after 50,000 loading cycles [26].

**Fig. 1:**
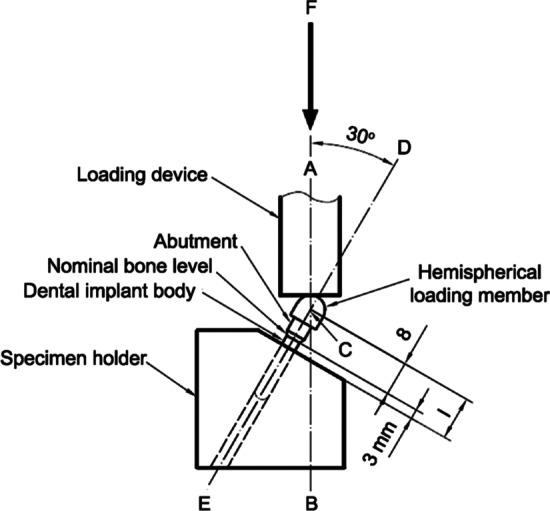
Fatigue test setup according to ISO 14801 [27]

The International Organisation for Standardization (ISO) initiated a method for implant fatigue test protocol in 2003; the latest version of the protocol was revised in 2016. This international standard simulates the cyclic loading of a dental implant under a “worst case” application, which is the most useful protocol to compare implant performance. The implant fixture protrudes from the supporting resin by 3 mm, representing vertical bone loss surrounding the fixture. The fixture is angled at 30° to the longitudinal axis, resulting in stress by the vertical and horizontal loads. Loading frequency should be limited to no more than 15 Hz. Mechanical testing of dental implants according to ISO 14801 recommendations may provide standard comparable data for the mechanical evaluation of implants on the market (Fig. 1) [27].

A static compression test is a standardized method to evaluate the behaviour of material under compressive load. Many studies have undertaken the compression test according to international standard ISO 14801 [28, 29]. A computer monitors the applied force continuously to the specimen with a constant speed of 1.0 mm/min. The lowest load which could break any component of the system is the ultimate strength of the system [29].

Most research on abutment-implant joint stability has focused on the diverse variables that might impact the screw preload values before and after dynamic loading. Joo-Hee Lee et al. studied the removal torque changes relative to abutment screw length [30]. Benjaboonyazit et al. evaluated removal torque change of a combined cone and octalobule index implant-abutment connection after mechanical cyclic loading [26]. Paepoemsin et al. measured the removal torque of three different abutment screws [31]. However, none of these studies compared the mechanical properties of customized titanium abutments with hybrid zirconia abutments.

The purposes of this study were to compare the removal torque values after mechanical cyclic loading and bending moment after static compression testing of the customized titanium abutment comparing with prefabricated and hybrid abutments.

## Materials and methods

This study model was set according to the international standard fatigue test (ISO 14801:2016), which is the standardized method to evaluate a fatigue test under the worst-case condition. Sixty implants with diameters of 4.2 mm. and lengths of 10 mm. (NOVEM DENTAL IMPLANT SYSTEM, Novem Innovations, Thailand) were embedded individually in epoxy resin block (CHOCKFAST® ORANGE, Shannon Industrial Estate, Ireland) at 3 mm. above the level of the upper rim of the resin block to represent the worst case.

**Fig. 2:**
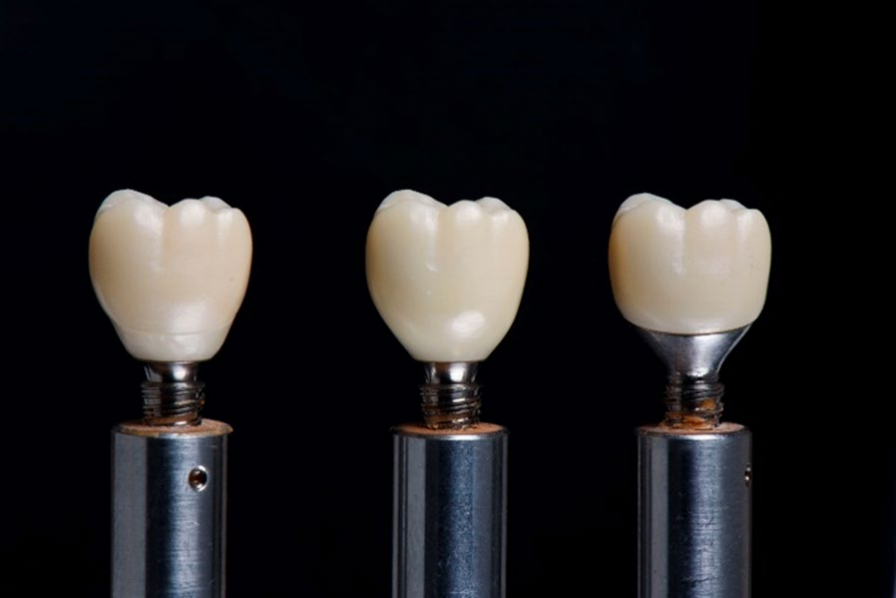
Hybrid zirconia abutment (left), Straight titanium abutment (middle), Customized titanium abutment (right)

All of the models were divided into three groups equally: Straight titanium abutment, Hybrid zirconia abutment, and Customized titanium abutment. All abutments were created by the implant manufacturer; the abutment connection was also produced with the same process. For the straight titanium abutment group, zirconia crowns were connected directly to the prefabricated straight titanium abutments. For the hybrid zirconia abutment group, zirconia crowns were connected to zirconia substructures which were made in the same procedure as the crown. The zirconia substructure provided the emergence profile of the restoration. For the customized titanium abutment group, zirconia crowns were connected to customized titanium abutments with margin and emergence profiles conformed to the zirconia substructures of the hybrid abutment group (Fig. 2).

Monolithic zirconia crowns (Cercon® ht, Dentsply Sirona Prosthetics, USA) were designed in the lower molar anatomy with screw holes using CAD application (3Shape Dental System™, 3Shape A/S, Denmark). The emergence profile of crowns was adapted according to the abutment design in each group. All zirconia crowns were machined and fired according to the manufacturer’s recommendations (inLab MC X5, inLab Profire, Dentsply Sirona, Germany).

Surface treatment before cementation was sandblasted with 50 µm alumina particles; 0.1 MPa for zirconia and 0.25 MPa for titanium. Then, all abutments and restorations were ultrasonically cleaned with isopropyl alcohol for 3 min and then dried. Dual-cure resin cement (PANAVIA™ V5, Kuraray Europe GmbH, Germany) was cemented between parts according to the manufacturer’s recommendations.

The abutments were attached to the implant fixtures in each group. The abutment screw was tightened according to the manufacturer’s recommended load (30 NCM) with a digital torque gauge (BTGE-G, Tohnichi America, USA). To reduce the settling effect, after 10 min, all abutment screw specimens were retightened at the same load (30 Ncm) and left unloaded for 10 min.

### Mechanical cyclic loading test

**Fig. 3:**
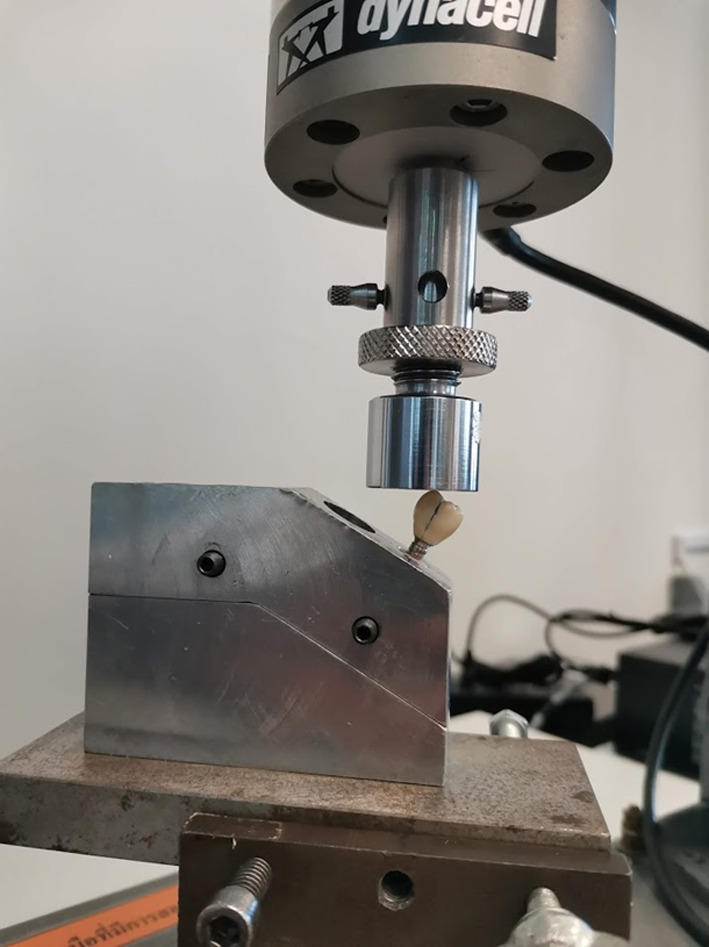
Sample set at a 30-degree angle to the loading direction according to ISO 14801

For each group of abutments, all samples (45 implants) were categorised into 3 groups. The positive control, 5 implants for each group, were measured the initial removal torque of the abutment screws using a digital torque gauge. The mean initial removal torque value were used as baseline value. The remaining samples underwent mechanical cyclic loading using a universal testing machine (ElectroPuls™ E1000, Instron Thailand, Thailand), which delivered a dynamic loading force, cycling between 15 and 250 N with a frequency of 15 Hz. The samples were set in the 30 ± 1-degree angle to the loading direction. The loading force was applied in a vertical axis through the load centre (Fig. 3).

Each remaining set underwent different numbers of mechanical loading cycles, as follows: 50,000 cycles and 1,000,000 cycles. After the mechanical cyclic loading, each set of implant samples was measured to evaluate the removal torque of the abutment screw using the digital torque gauge.

The removal torque values before and after cyclic loading in all groups were recorded and calculated for percentage loss of removal torque values (RTVs of the control group).

### Static compression test

All groups of abutment types (15 implants, 5 implants for each group) underwent the static compression test using a universal testing machine. The machine applied load at a rate of 1 mm/min until the specimen fracture or reached a maximum displacement at 6 mm. On the occlusal surface, 0.5 mm aluminium foil was applied to distribute the vertical load to the occlusal surface.

The applied load and displacement data of the load cell were collected in real time with wave matrix software. The maximum force before failure was defined as load-bearing capacity. To define the fracture resistance of the model, the bending moment for each group was calculated by multiplying the distance of load contact to the upper rim of the resin block in abutment axis. However, all models were created with the same design, so they had equal distance.

### Statistical analysis

The removal torque values were measured for all groups. Statistical analysis was performed using one-way analysis of variance (one-way ANOVA) and two-way analysis of variance (two-way ANOVA) for the overall effect of the types of abutment on the removal torque values of the abutment screw. A multiple comparison analysis was conducted using Dunnett’s T3 Test with SPSS 20 (IBM SPSS, USA). All samples’ removal torque values in mechanical cyclic loading testing underwent for a power analysis using G*Power (HHU, Germany) [32, 33]. A power (1 − *β*) is 0.92 for mechanical cyclic loading test.

Load-bearing capacity was analysed statistically with one-way analysis of variance (one-way ANOVA) to compare the effect of the types of abutment on the load-bearing capacity of each types of abutment. Multiple comparison analysis was conducted using the post hoc Tukey’s HSD test with SPSS 20.

## Results

According to the cyclic loading test, the removal toque value data were normally distributed (Shapiro–Wilk test). A summary of all cyclic loading groups is shown in Table 1.

**Table 1:** Comparison of mean removal torque values (Ncm) for the abutment screws in all groups

Abutment/Number of cycles	Mean removal torque value (Ncm) ± SD	% of mean removal torque value loss compared to the initial	Post hoc Dunnett’s T3 test of mean removal torque (*P*-value)							
			ST/0	HZ/0	CT/0	ST/50,000	HZ/50,000	CT/50,000	ST/1,000,000	HZ/1,000,000
ST/0	28.64 ± 0.63	–								
HZ/0	28.43 ± 0.52	–	1.000							
CT/0	27.44 ± 0.86	–	0.621	0.769						
ST/50,000	23.94 ± 1.70	19.63%	0.050	0.062	0.158					
HZ/50,000	23.23 ± 1.16	22.38%	0.003	0.005	0.011	1.000				
CT/50,000	22.46 ± 1.64	22.17%	0.013	0.017	0.030	0.991	1.000			
ST/1,000,000	23.71 ± 1.11	20.79%	0.004	0.006	0.019	1.000	1.000	0.988		
HZ/1,000,000	21.82 ± 5.60	30.29%	0.562	0.591	0.748	1.000	1.000	1.000	1.000	
CT/1,000,000	22.42 ± 2.46	22.39%	0.076	0.088	0.157	0.999	1.000	1.000	0.999	1.000

*ST* straight titanium abutment, *HZ* hybrid zirconia abutment, *CT* customized titanium abutment

**Fig. 4:**
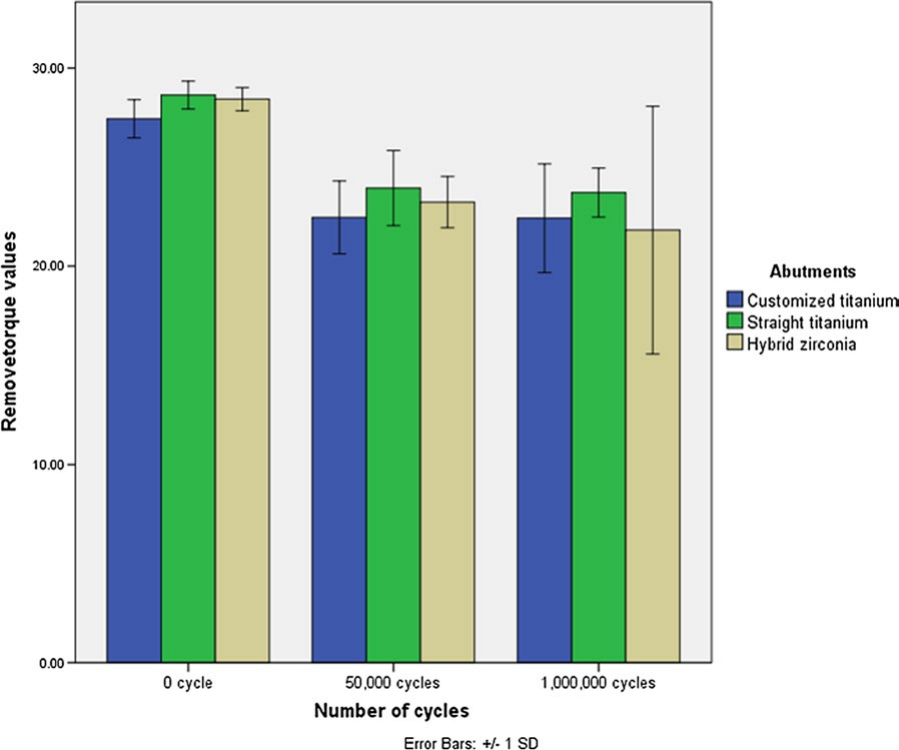
Comparison of the mean removal torque value between types of abutments at 0 cycle (initial), 50,000 cycles, and 1,000,000 cycles. The zero-cycle group is significantly higher than other groups. (*P*-value < 0.001)

The mean initial removal torque value of the control groups is significantly higher than 50,000 cycles and 1,000,000 cycles (*P* < 0.001). The mean removal torque value of straight titanium abutment is higher than other abutments, but without any significant difference at 50,000 cycles (*P* = 0.414) and at 1,000,000 cycles (*P* = 0.753). The post hoc comparison of the mean removal torque value between types of abutments and cycles is shown in Table 1 and Fig. 4. No screw loosening was found in any group.

**Fig. 5:**
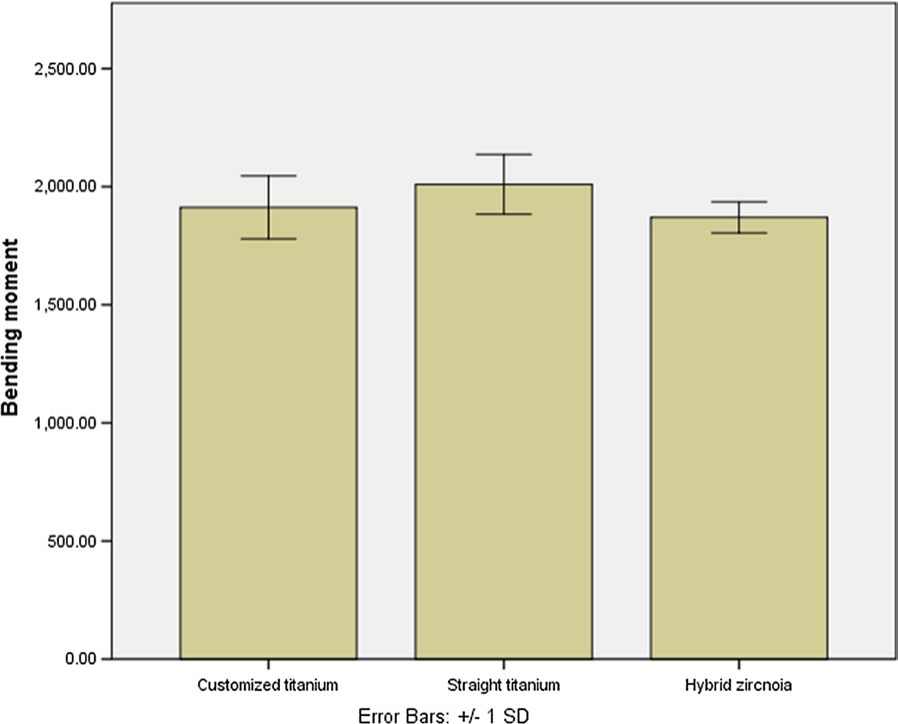
Comparison of the mean of bending moment between abutment types. No statistically significant difference found (*P*-value > 0.05)

The bending moment of straight titanium abutment (2009.92 Ncm) was not significantly different compared with hybrid zirconia abutment (1870.03 Ncm) and customized titanium abutment (1262.29 Ncm) (P > 0.05) (Table 2, Fig. 5). No zirconia restoration fracture was found in any group.

**Table 2:** Mean maximum forces before failure (N) and bending moment in all abutment groups

Type of abutment	Mean maximum forces before failure (*N*) ± SD	Bending moment (Ncm) ± SD	Post hoc Tukey’s HSD test of multiple comparison of mean removal torque (*P*-value)	
			Straight titanium abutment	Hybrid zirconia abutment
Straight titanium abutment	1326.68 ± 74.83	2009.92 ± 113.37		
Hybrid zirconia abutment	1234.34 ± 38.81	1870.03 ± 58.80	0.164	
Customized titanium abutment	1262.29 ± 78.45	1912.37 ± 118.85	0.386	0.862

## Discussion

Customized abutments allow for individual emergence profile. According to the 1-year prospective study, CAD/CAM abutments could maintain normal form of dental papilla significantly compared with casting custom abutment [34]. CAD/CAM technology improve the efficiency of customized abutment.

This study focused on abutment types with the same implant-abutment connection design. An in-vitro test was designed to demonstrate the impact of abutment types on abutment stability. The stability of abutments was investigated after cyclic loading at 50,000 cycles and 1,000,000 cycles. Previous study found that the removal torque values showed significant change after 50,000 and 1,000,000 cycles [26, 31]. The removal torque values represented the remaining clamping force of the implant-abutment connection. The results of our study showed that the removal torque values were not significantly different between 50,000 cycles and 1,000,000 cycles. Benjaboonyazit and colleagues studied the removal torque values in different loading cycles and reported similar result [26].

In the control group of this study, the removal torque value ranged between 4.53 and 8.53% loss after tightening the screw. Previous study found preload loss between 2 and 10%. This is in agreement with other reports that suggested it could be a result of the settling effect [5, 7]. The assumption of the settling effect results from flattening of a rough spot on contacting surfaces. Wear of contact surface occurs after screw tight. This results in lower removal torque compared with initial torque [5].

Low modulus of elasticity could absorb stress and have a damping behaviour to loading force. High modulus showed low deformation, which means the force can be transferred through the material. Restorations with higher modulus could transfer more loads to abutments [35]. Zirconia is widely used due to its colour and sufficient mechanical strength. Young’s modulus of zirconia (210 GPa) is higher than titanium (110 GPa) [36]. With this property, zirconia shows less shock absorption effect than titanium [35]. The occlusal loading force that directs to the implant-abutment interface could influence stability [5]. Consequently, zirconia abutments may show less implant-abutment stability than titanium abutments. In addition, type of luting material also affects shock absorbing capacity [35]. According to our study, a customized titanium abutment has more volume of titanium than other abutments, but the removal torque values are not different significantly compared with other abutments. Modulus property of abutment and luting cement might influence implant-abutment stability, but it does not seem to affect removal torque values or bending moment. Another study reported that the titanium abutment has a higher bending moment than zirconia abutment [37]. However, using a secondary metallic component could empower the implant-abutment stability of zirconia abutment [38, 39]. In this study, a hybrid abutment is a two-piece abutment containing metallic connection. Thus, the bending moment of the hybrid abutment was not significantly different compared with other groups. With titanium connection material, the bending moment is not different, regardless of the type of abutment.

Customized titanium abutments and hybrid abutments are made with CAD/CAM technology; the structure beyond the connection can be modified with less limitation. According to previous study, there was no different bending moment between anatomical abutment and straight abutment. However, it was found that the longevity of anatomical abutment after fatigue test was lower than the straight abutment group [40]. In this study, both a customized titanium abutment and hybrid abutment were created with anatomical design; the connection for abutments was made with the same manufacturing. The bending moment was not different between anatomical and straight abutment. Survival rate of customized abutments should be investigated further in long term studies.

Low-temperature degradation or aging of zirconia affect strength of the material by phase transformation. Decreasing strength is a result of the increasing proportion of the monoclinic phase [41]. Water or moisture accelerates this transformation [42]. Many studies have designed wet condition methods regarding aging to simulate clinical situations [43–45]. However, some studies still designed a dry condition method [26, 46]. Lee and colleagues suggested that saline could increase the crack propagation of zirconia, but not show any effect on failure [47]. According to previous study, our study was performed under dry condition. The effect of aging zirconia did not influence the results of our study.

Implant-abutment selection should be considered according to the biocompatibility, mechanical stability, and aesthetics [48]. Implant-abutment stability influences the long-term success of dental implant treatments. As found in our study, type of abutment might not affect implant-abutment stability. Thus, clinicians could select any customized abutment or prefabricated abutment regardless of mechanical stability. However, it is recommended to place the cement margin of abutments as shallow as possible to prevent submucosal cement remnants. Customized abutments could place individual cement margin line related to scalloped soft tissue [14, 49]. Customized abutment material should be considered meticulously in aesthetic area. Anodized titanium abutment or zirconia abutment could achieve better aesthetic outcome than unanodized titanium abutment [50]. According to the 4th EAO Consensus, there were no significant difference between titanium and zirconia abutment [51].

Implant-abutment connections are also taken into considerations. The implant system used in this study (NOVEM DENTAL IMPLANT SYSTEM, Novem Innovations, Thailand) has a cone-index connection with 5-degree taper, titanium alloy grade 5 retaining screw which are the implant abutment designed and screw material mostly used by the other implant systems in the market. However, the abutment screw torque depends on the implant system. Even for the same tapered joint, the removal torque after mechanical cyclic loading is expected to be different depending on the screw type shape, and implant-abutment connection design [52].

A study from Katsuta and Watanabe [52] on abutment screw loosening of dental implant after cyclic torsional loading showed 9.9–13.5% reduction of removal torque in the implant system with cone-index implant-abutment connection, while in our study show 20.79% in ST group, 30.29% in HZ group, and 22.39% in CT group. This different of removal toque may be due to our worst scenario cyclic loading setting.

The standard error of hybrid abutment appears to be large for the removal torque value at 1,000,000 cycles, this may be result from the two layers of cement between titanium base and substructure and between substructure and zirconia crown.

The superstructure used in this study is zirconia which is presently more popular material used for implant prosthesis. However, with different prosthesis material such as porcelain fused to metal, all metal prosthesis, the results may be different. The further studies are required.

This analysis involves in-vitro study. However, future clinical study should be investigated. A varied implant-abutment connection designed, clinical environment such as temperature variations, oral fluid, and parafunctional habit might generate different results compared with this study. Within the limitations of this study, the power (1 − *β*) of static loading test was low. Larger sample sizes should be investigated in future study.

## Conclusion

Within the limitation of this in-vitro study, it could be concluded that customized titanium abutments, hybrid abutments and straight titanium abutments are not significantly different in terms of removal torque values after fatigue testing. The bending moment between types of abutment was not significantly different.

## Data Availability

The data sets used and/or analyzed during the current study are available from the corresponding author on reasonable request.
